# Importance of long-term monitoring of patients with breast reconstructions: a case of 10-year cancer recurrence

**DOI:** 10.1080/23320885.2021.2011288

**Published:** 2021-12-13

**Authors:** Sarah Hardwick, Sanjeev Hariparsad, Nakul Kain, Charles M. Malata

**Affiliations:** a Cambridge Breast Unit, Addenbrooke’s University Hospital, Cambridge, UK; b Plastic & Reconstructive Surgery Department, Addenbrooke’s University Hospital, Cambridge, UK; c Postgraduate Medical Institute at Anglia Ruskin University, Cambridge & Chelmsford, UK

**Keywords:** Breast cancer recurrence, breast cancer monitoring

## Abstract

We report a case of breast cancer recurrence in a 41 -year old female ten years post mastectomy, and two years post tertiary DIEP flap reconstruction. Reconstructed patients, especially those with aggressive cancers, must be informed of long term risk of recurrence and monitored long term following mastectomy and reconstruction.

## Introduction

Breast cancer has a long term recurrence risk with a local recurrence rate in a mixed cohort of wide local excision and mastectomy patients of 1.0–1.5% per year for 15–20 years [1]. Whilst mastectomy patients are considered to have the lowest risk, the lifetime risk of recurrence remains in the range of 2.3–5.0% [2]. Cancer histology, stage, grade, lymph node status, commercially available gene expression genomic classifiers, and clinical factors are all used to predict a patient’s long term risk of recurrence. It has been well established in patients undergoing ablative surgery that the majority of breast cancers recur within the first 5 years [3–6]. Risk of recurrence increases with higher tumour grade, positive nodal status, and certain histological subtypes; higher rates of local recurrence are reported among Her2 positive, triple negative, and ductal carcinoma *in situ* containing histology in patients undergoing breast conserving surgery [7]. Local recurrence is rare in patients who undergo mastectomy for ductal carcinoma [8–10]. After mastectomy, lymph node status and tumour size are the dominant risk factors for local recurrence [8], with an increased risk demonstrated in one retrospective study in young patients, those with ductal carcinoma tumour subtypes, high grade tumours, and evidence of microinvasion [11]. Recurrences more than 10 years after oncological surgery are especially rare [12].

In recent decades, outcomes of breast cancer management have focused not only on surgical and oncological clearance but also on the aesthetics of partial or total breast reconstruction. Whilst reconstruction has greatly improved patient quality of life and psychological outcomes following oncological breast surgery, it may mask clinical and radiological detection of breast cancer recurrence. Fat necrosis can occur secondary to hypoxia in flaps used in autologous tissue based reconstruction. Post operative fat necrosis occurs in 6–18% of deep inferior epigastric perforator flap (DIEP) and 10–24% of transverse rectus abdominis musculocutaneous flap (TRAM) reconstructions [13]. On clinical examination, the physical manifestation of necrosis is a firm palpable mass that may mimic a recurrent tumour. On imaging, it can appear as a smooth bordered lucent mass resembling a cyst or, less commonly, as pleomorphic calcifications, which render its differentiation from recurrent tumour more difficult [14]. A biopsy is mandatory to investigatesuspicious findings on imaging or physical examination [15].

We herein describe a case of breast cancer recurrence in a patient post DIEP and autologous fat grafting (AFG). Our patient presented with recurrent ductal cancer 10 years after mastectomy in the DIEP-reconstructed breast and 18 months after AFG. Her case emphasises the importance of post-operative monitoring in patients with high grade invasive cancers, and the role oncological physicians, breast surgeons, and plastic surgeons play in concert communicating ongoing long term risks to patients post reconstruction. Plastic surgeons may see patients later in their treatment journey than the oncological breast surgeons for secondary (delayed) or tertiary breast reconstruction, and therefore have an important role in emphasising both the importance of self-examination and adherence to local monitoring protocols post operatively, even after curative surgical, radiological, and oncological treatments are complete.

## Case presentation

**Figure 1.**
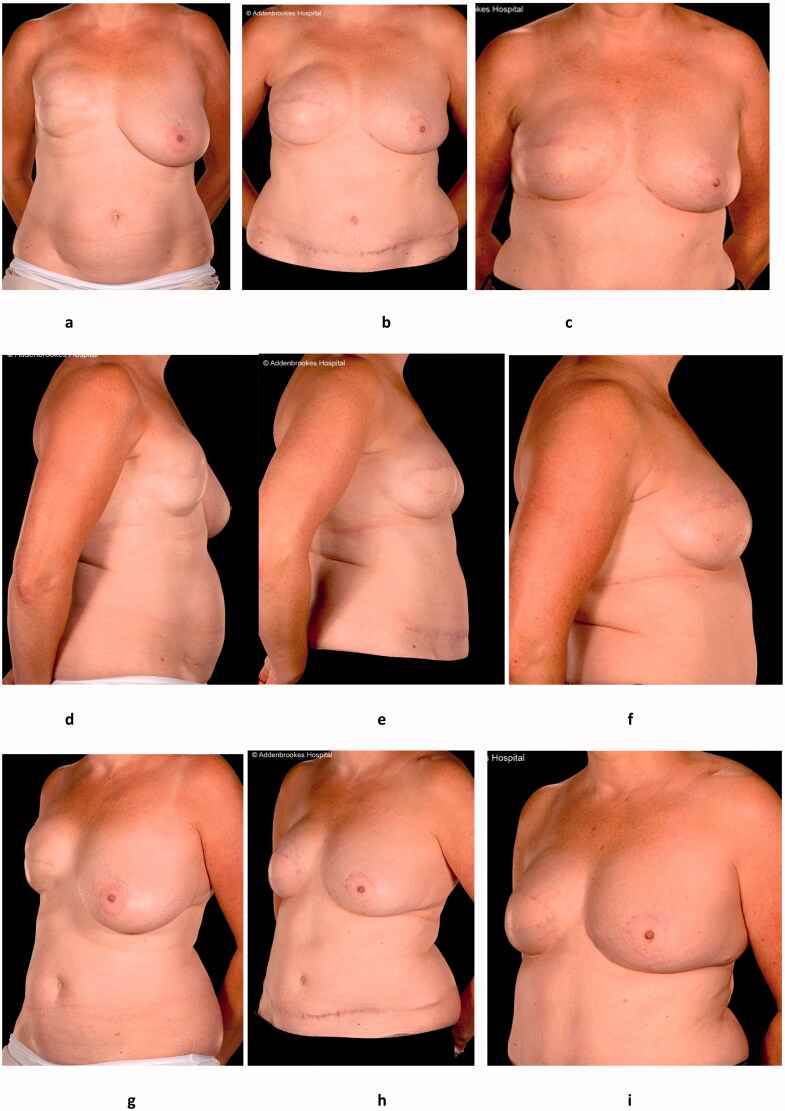
Clinical photographs of the AP, right lateral and left oblique views of the patient before salvage surgery (a, d, g), after the DIEP flap (b, e, h) and after fat grafting (c, f, i) to the volume deficit and the contour deficiency especially in the take-off superiorly.

A 41-year-old woman was diagnosed with right-sided breast cancer in 2003. She underwent skin-sparing mastectomy and axillary clearance, with immediate breast reconstruction (IBR) with an expandable implant (hereafter referred to as an expander). The histopathological results revealed an 8.4 mm grade 2 invasive ductal carcinoma with associated intermediate grade ductal carcinoma *in-situ*. The tumour was oestrogen, progesterone and HER2/NEU receptor positive, with clear resection margins and no vascular invasion. One out of 29 axillary lymph nodes examined was positive, giving her a Nottingham Prognostic Index score of 5.7. She therefore received postoperative chemotherapy and radiotherapy, as well as tamoxifen and anastrozole therapy which were completed in 2009. However, prior to receiving radiotherapy, an expander-to-implant exchange had to be performed earlier than planned due to spontaneous deflation of the expander. Despite this the patient remained unhappy with her reconstruction and was therefore referred to the plastic surgery service in 2010. Given her suboptimal reconstruction combined with radiation-induced capsular contracture, a totally autologous conversion to a free flap was undertaken [16]. This salvage surgery comprised total capsulectomy with implant removal and tertiary [16] reconstruction with a DIEP flap. A simultaneous contralateral balancing mastopexy was also performed (Figure 1: pre-salvage, post-salvage and post-fat grafting appearances). Histopathological analysis of the capsulectomy specimen, the mastectomy scar, and an incidental internal mammary lymph node showed no evidence of malignancy. The breast tissue from the contralateral mastopexy showed no abnormality.

Although symptoms from previous capsular contracture were resolved, the reconstructed breast was smaller than the opposite breast despite the simultaneous balancing surgery (Figure 1(b,e,h). In 2011, she therefore underwent Coleman fat transfer from the abdomen to the right DIEP reconstruction, both into the deep and superficial layers of the DIEP flap. This was repeated 6 months later in 2012. In our institution, post-operative autologous tissue flap patients are counselled to perform monthly self-examination. Post mastectomy patients have routine follow up with the breast oncology team at least bi-annually for two years after oncological surgery, with increased frequency at patient or physician’s discretion, in line with NICE guidance [17]. They additionally have planned follow up with the plastic surgery team 1 year after reconstructive surgery. However routine imaging, such as screening mammography, is not routinely performed, in line with guidance from the Royal College of Radiologists [18]. Therefore, as her two year term for follow up clinical examination with her oncological breast surgeon was complete, she had no routine follow up beyond her one year post- reconstruction appointment with the plastic surgery team, and no screening mammogram. In 2013, 18 months from her first fat grafting procedure and 10 years from the mastectomy, she noted two small lesions on self-examination in her right reconstructed breast– one in the upper inner quadrant and the other in the lower outer quadrant.

She was referred for urgent assessment to her oncological breast surgeon. As on clinical examination her new breast lumps were clinically different from the cysts/fat necrosis that can be expected following fat grafting, the patient underwent ultrasound guided biopsies of both lesions. These revealed a grade two invasive ductal carcinoma with oestrogen and HER2/NEU receptor positivity: recurrence of her original tumour. She underwent resection of the recurrent tumours, including the DIEP flap without further reconstruction. She remains well and disease-free 9 years later.

## Discussion

While there is ample recommendation on when to commence follow up of imaging surveillance following mastectomy (Table 1) there is scant advice on ipsilateral imaging surveillance. Decisions about when to step down or reduce frequency of clinical examination and follow up are often left to the discretion of treating physicians. In a review of 18 publishing bodies [23] 13/18 did not recommend image screening the ipsilateral breast for recurrence, 5/18 provided no guidance, and only the United Kingdom Royal College of Radiologists recommended ipsilateral annual mammogram in the special case of high-risk patients; for example, extensive high grade DCIS close to a margin in autologous tissue reconstructed breasts [18]. Therefore late ipsilateral mastectomy site cancer recurrence, with or without breast reconstruction, may go undetected clinically until it is locally advanced or metastatic.

**Table 1.** International guidance from major regulatory bodies in the US, UK, and Europe on recommended post-operative clinical and radiological examination frequency mastectomy for breast cancer.

Guideline	History and clinical examination	Imaging onset: screening mammogram	Imaging frequency	Imaging frequency reduction and termination
ACR [19]	No recommendation	Contralateral 6–12 months post radiotherapy	Annual	Institutions to decide when to return to routine breast cancer screening
ACS-ASCO [20]	3–6 Months for first 3 years, Every 6–12 months for years 4–5, then annually	Contralateral >6 months post radiotherapy	Repeat every 6–12 months and reduce to annual if MMG stable	No recommendation
ESMO [21]	Every 3–4 months for first 2 years, Every 6 months years 3–5, then annually	Contralateral: onset timing not specified	Annual	No recommendation
NICE [17]	Regular follow up appointments as stipulated by physician or patient	Not specified	Annual	After 5 years if >/= NHS BSP screening age, frequency thereafter not specified
NCCN [22]	4–6 Months for first 5 years then annually	Contralateral 6–12 months after RT	Annual	No recommendation
RCR [18]	Not specified	Contralateral: onset not specified. Ipsilateral if high risk and recipient of autologous tissue reconstruction	Annual	Consider reduced frequency age 50 and cessation age 75

Abbreviations: ACR: American College of Radiologists; ACS: American Cancer Society; ASCO: American Society of Clinical Oncology; ESMO: European Society of Medical Oncology; NICE: National Institute of Clinical Excellence; NHS BSP: National Health Service Breast Screening Programme; NCCN: National Comprehensive Cancer Network; RCR: Royal College of Radiologists.

Locoregional recurrence risk in the reconstructed breast is similar to the risk after mastectomy alone, and reconstruction is not considered an oncologically provocative additional risk [24,25]. A retrospective review of 554 mammograms in 256 women who underwent TRAM flap reconstruction concluded that annual mammography in all autologous tissue based breast reconstructions yielded low additional detection rate of clinically occult malignancy, and concluded routine surveillance would not be beneficial [26]. The onus is therefore on individual treating physicians to proactively ensure recurrence is detected. This patient’s recurrence, occurring 10 years after primary oncological surgery, demonstrates the ongoing risk, and the role that plastic surgeons, who may see patients later in their recovery for delayed reconstruction, can fill in emphasising this ongoing risk. Whilst evidence to date argues against routine imaging screening in cancer patients who undergo ipsilateral breast reconstruction, late recurrence is still well-documented; Case reports of late recurrence post reconstruction have been published following DIEP flaps at 3 and 9 years post mastectomy in patients treated for intraductal carcinoma [15].

## Conclusion

As long term recurrence is a well-established, if rare, and ongoing risk, the temporal relationship between reconstruction, fat grafting and recurrence will be complex. Based on cancer biology, we may assume that this patient’s recurrence was already present in a subclinical context, and that the imaging and additional examinations that followed late reconstruction and fat grafting simply expedited clinical detection. Cases such as ours [15] where interval follow up for delayed reconstruction facilitated detection of cancer recurrence, suggest an extension of the time window for repeat clinical examination in high risk patients may be warranted. Indeed this would likely be in accord with patients’ wishes; In a survey of breast cancer survivors, 56/84 indicated they would like to attend lifelong follow up [27]. However, as this is not universally clinically feasible, communication with patients on long term risk and the importance of self-examination is paramount. Identification of high risk sub-groups of mastectomy patients receiving delayed or salvage breast reconstruction could facilitate more personalised, extended post-operative follow up protocols. In the interim, plastic surgeons in the UK and internationally may wish to take up the Royal College of Radiologists’ guidance to perform annual mammograms for high risk patients with autologous tissue based reconstructions [18].

## Report limitations

This patient was monitored according to the UK NICE [17] guidelines for follow up post oncological surgery and Royal College of Radiologist guidelines [18] for imaging post autologous tissue based reconstruction. She received annual contralateral mammogram screening on the non-resected breast, but as she was not high risk for recurrence based on her clear resection margins, she did not receive ipsilateral screening mammograms following her DIEP flap. Her oncological surgery follow up was bi-annual for the first two years following mastectomy, and had long ceased by the time of her reconstructive surgery seven years later. Following recurrence, she was followed by the oncological breast surgeons biannually for a further two year period.
